# Iron-related toxicity of single-walled carbon nanotubes and crocidolite fibres in human mesothelial cells investigated by Synchrotron XRF microscopy

**DOI:** 10.1038/s41598-017-19076-1

**Published:** 2018-01-15

**Authors:** Francesca Cammisuli, Silvia Giordani, Alessandra Gianoncelli, Clara Rizzardi, Lucia Radillo, Marina Zweyer, Tatiana Da Ros, Murielle Salomé, Mauro Melato, Lorella Pascolo

**Affiliations:** 1 Department of Medical, Surgical, and Health Sciences, University of Trieste, 34149 Trieste, Italy; 2 Department of Chemistry, University of Turin, Turin, Italy; 3 Elettra - Sincrotrone Trieste, Basovizza, 34149 Trieste, Italy; 4 Department of Chemical and Pharmaceutical Sciences, University of Trieste, 34149 Trieste, Italy; 5 European Synchrotron Radiation Facility, 38000 Grenoble, Cedex 9 France; 6 Institute for Maternal and Child Health, IRCCS Burlo Garofolo, 34137 Trieste, Italy

## Abstract

Carbon nanotubes (CNTs) are promising products in industry and medicine, but there are several human health concerns since their fibrous structure resembles asbestos. The presence of transition metals, mainly iron, in the fibres seems also implicated in the pathogenetic mechanisms. To unravel the role of iron at mesothelial level, we compared the chemical changes induced in MeT-5A cells by the exposure to asbestos (crocidolite) or CNTs at different content of iron impurities (raw-SWCNTs, purified- and highly purified-SWCNTs). We applied synchrotron-based X-Ray Fluorescence (XRF) microscopy and soft X-ray imaging (absorption and phase contrast images) to monitor chemical and morphological changes of the exposed cells. In parallel, we performed a ferritin assay. X-ray microscopy imaging and XRF well localize the crocidolite fibres interacting with cells, as well as the damage-related morphological changes. Differently, CNTs presence could be only partially evinced by low energy XRF through carbon distribution and sometimes iron co-localisation. Compared to controls, the cells treated with raw-SWCNTs and crocidolite fibres showed a severe alteration of iron distribution and content, with concomitant stimulation of ferritin production. Interestingly, highly purified nanotubes did not altered iron metabolism. The data provide new insights for possible CNTs effects at mesothelial/pleural level in humans.

## Introduction

Nanotechnology has become one of the most promising fields in science and technology, with increasing number of applications in materials science, sensing, bioimaging, medicine and biology^1–3^.

Many different nanomaterials (both organic and inorganic) are currently under investigation as therapeutic, diagnostic agents or, more frequently, new drug delivery systems^4–6^, and the related environmental, health and safety issues have been given increasing attention.

Among other materials, since the beginning of the 21^st^ century, the unique properties of carbon nanotubes (CNTs) made them very promising candidates in nanomedicine for biomedical applications, not only for drug delivery and gene therapy, but also for tissue regeneration and diagnostic biosensoring^7–9^. Thanks to their unique surface area, excellent chemical stability, and rich electronic polyaromatic structure, they are able to absorb or conjugate with a wide variety of therapeutic molecules (drugs, proteins, antibodies, DNA, enzymes, etc.) and they have been proven to be an excellent vehicle for drug delivery by penetrating into the cells directly and keeping the drug intact without metabolism during transport through the body^10,11^. Although CNTs characteristics are associated with highly desirable properties, the drawback is that the state of knowledge regarding their possible unwanted side effects is still limited^9,10^. This is particularly relevant since exposure of general population to this material is expected to increase in the future. The potential toxicity and exposure risks are not only for future patient’s safety, but also, and mainly, for workers exposed to health hazards during CNTs synthesis and manipulation^12,13^.

The use of CNTs, particularly in industrial applications, is currently considered with apprehension because of their yet undefined safety profile and especially given their fibrous structure that might cause asbestos-like pathology in the lung and mesothelium^14,15^. Recently the IARC has included some multi-walled CNT (MWCNTs) in the list of carcinogens, in the same category of asbestos^16^. Some reviews summarized most of the studies demonstrating the similar toxic effects of CNT and asbestos fibres both *in vitro* and *in vivo* models^17–20^. One of the first alarming report comes from Takagi A. *et al*. and describes an *in vivo* study where the single injection of long and short MWCNTs into the peritoneal cavity of mice induced the development of mesothelioma^21^. More recently, other authors demonstrated that multi-walled CNTs can cause also pleural mesothelioma in wild type mice^22^. The length-dependent response to CNT demonstrated by many studies, both *in vivo* and *in vitro*, suggests that the longest fibres are those implicated in mesothelioma development as well as in inflammatory responses^19,23–25^.

In general, both the specific chemical composition of nanomaterials and the surface properties are implicated in nanofibres toxicity. Considerable evidences suggested that reactive oxygen species (ROS) such as hydrogen peroxide (H_2_O_2_), superoxide anion (O_2_^−^) and hydroxyl radical (HO^•^), together with the reactive nitrogen species (RNS), can be generated directly by the fibres themselves or indirectly, through interactions with inflammatory cells^26,27^.

The presence of transition metals in the fibres and the ability of the latter to attract metal particles are central processes to explain carcinogenic effects of asbestos. The presence of both ferrous (Fe^2+^) and ferric (Fe^3+^) forms is considered to be the cause for the genotoxic and cytotoxic responses after asbestos fibres deposition^28^. Among commercially used asbestos fibres, crocidolite and amosite asbestos contain 20–30% iron by weight and are considered the most carcinogenic^28^.

Metals (i.e. iron, nickel, cobalt and molybdenum) are used as catalysts in the synthesis of CNTs, to promote the CNTs growth. Typically the purification is not efficient enough to remove all the catalysts, thus the resulting samples are not completely metal-free. The presence of metals, particularly iron, has been recognized as a mediator of fibre toxicity and carcinogenicity in diverse preparations of carbon nanotubes^28^. In some CNT studies bioavailable iron has been associated with increased oxidative stress^29^ and inflammatory responses^30^.

In an intriguing study, Bussy and co-workers demonstrated that numerous iron-based nanoparticles are present on the sidewalls of CNTs, mainly originated from the decomposition of iron pentacarbonyl during CNT synthesis; apparently these particles rapidly detach upon endocytotic internalization of nanotubes in macrophages, becoming quickly available for oxidative stress reactions. In that study a synchrotron based microscopy approach (SR-XRF) was applied similar to that of the present manuscript^31^.

In the case of asbestos fibres, it has been demonstrated that the relation between asbestos pathogenicity and iron is not only due to the presence of a reactive metal on fibre surfaces, but also, and more importantly, to the fact that asbestos causes a real alteration in the iron homeostasis of the cells, interacting with the fibres^32–34^. In line with these observations, by using synchrotron radiation X-ray fluorescence microscopy (SR-XRF) we previously showed that iron and other elements are involved in the response of lung tissue to asbestos, in particular we revealed that the asbestos causes a continuous mobilization of iron from the surrounding cells, mainly alveolar macrophages^35,36^.

In the present work we hypothesize that CNTs may alter iron metabolism in biological systems as asbestos does, and we also wonder whether iron changes are proportional to the content of iron impurities of CNT preparations. This mechanism may be maximally relevant in the pleural tissues: we therefore used an *in vitro* model of mesothelial cells (Met-5A). Synchrotron based soft X-ray imaging (absorption and phase contrast images) and X-ray Fluorescence (SR-XRF) microscopy were performed to evaluate the effects and the iron concentration changes in cells exposed to asbestos (crocidolite), raw single-walled carbon nanotubes (R-SWCNT), as well as purified and highly purified single-walled carbon nanotubes (P-SWCNT and HP-SWCNT, respectively). Changes in iron metabolism were also investigated by assessing ferritin content of treated cells.

## Materials and Methods

### Crocidolite asbestos fibres

Crocidolite Asbestos UICC Standard fibres (SPI#02704-AB)^37^ were purchased from SPI Supplies Division, Structure Probe, Inc. (West Chester, PA 19381-0656, USA) and suspended in sterile phosphate buffered saline (PBS) at a concentration of 10 mg/mL. The fibres size parameters are reported in detail in Kohyama *et al*.^38^: they spanned from 0.5 to 100 µm in length and from 0.1 to 1.2 µm in width. The fibres were sonicated for 1 h and forcibly dispersed by using a 21-gauge needle attached to a 5–10 mL syringe.

FeSO_4_ heptahydrate was purchased from Sigma Aldrich (St. Louis, MO, USA). Ferrous sulphate solution was prepared by dissolving the pure powder in sterile H_2_O at final concentration of 80 µM.

### Carbon Nanotubes

Raw single-walled nanotubes (R-SWCNTs) produced by the HiPCO technique were purchased from Unidym®, Inc. (Lot no. R1912/R0513). The purified ones (P-SWCNTs) were prepared according to the protocol in Bonifazi *et al*.^39^. Briefly, they were obtained by treating pristine HiPCO SWCNTs with HNO_3_ and by oxidation with H_2_SO_4_–H_2_O_2_ at 45 °C for 1 h^39^. Highly purified SWCNTs (HP-SWCNTs) were obtained using the protocol reported by Flavin *et al*.^40^ Raw HiPCO SWCNTs were treated in HNO_3_ (7 M) for 4 h and subsequently refluxed in NaOH (2 M). Then the SWCNTs were subjected to a second oxidation step which consisted of refluxing in H_2_O_2_ 10% for 1 h followed by diluting/quenching with ice and allowing to stand for 12 h. Finally the remaining carbonaceous impurities were removed through a sodium hydroxide treatment, yielding highly purified SWCNTs.

Chemical and morphological characterization has been performed as routinely and already reported before^39–41^.

The main characteristics of carbon nanotubes used in this study are summarized in Table 1.

**Table 1:** Different type of SWCNT and their iron content.

Type SWCNT	Diameter	Length	Residual Fe Catalyst
R-SWCNT	~0.8–1.2 nm	~1000 nm	<30 wt%
P-SWCNT	~0.8–1.2 nm	~600 nm	<15 wt%
HP-SWCNT	~0.8–1.2 nm	~200–500 nm	<2 wt%

Just before use, SWCNTs (“raw” or pristine R, purified P and highly purified HP) and crocidolite fibres were suspended in serum-free cell culture medium (DMEM) at final concentration of 5 µg/mL. Nanomaterial suspensions were sonicated for 15 minutes under temperature-controlled conditions (+4 °C), with 15 seconds interruption every 5 minutes for rotating steps.

### Cell culture and treatments

Human mesothelial MeT-5A cells (ATCC) were maintained in Dulbecco’s modified Eagle’s medium (DMEM) containing 10% fetal bovine serum (FBS), L-glutamine 2 mM, 100 U/mL penicillin and 100 U/mL streptomycin at 37 °C in a 5% CO_2_ atmosphere. The cells were cultured in 75 cm^2^ Falcon flask for 2–3 days, then harvested by exposure to trypsin and transferred onto 24-well plates for viability tests by trypan blue exclusion assay or cultured onto silicon nitride (Si_3_N_4_) 100 nm thick membranes (Silson Ltd., Northampton, United Kingdom) contained in 24 multiwell plates for SR-XRF analysis^42,43^.

After 24 h half-confluent MeT-5A cells were treated for 24 h with 500 µL of medium containing SWCNTs (R, P, HP) and crocidolite fibres to obtain the final concentration of 5 µg/mL. Just before use, the nanofibres were sonicated in serum-free cell culture medium.

### Cell viability test in MeT-5A

Potential toxicity of crocidolite and SWCNTs (pristine, purified and highly purified) was evaluated on mesothelial cells (MeT-5A) by trypan blue exclusion dye test. The fibres cytotoxicity was tested for 24 h at concentrations ranging from 1 to 20 µg/mL. After completion of exposure time, the cells were rinsed twice with sterile phosphate-buffered saline (0.1 mM PBS, pH 7.4), then detached from the well by 0.25% trypsin (Sigma-Aldrich) containing 0.03% EDTA, pelleted (1200 rpm for 10 min) and resuspended in protein-free medium. Then, 10 μL cellular suspension was mixed gently with 40 μL of trypan blue in an Eppendorf tube and incubated for 5 min at room temperature. The cell suspension was counted in a Bürker chamber (HBG, Germany). Triplicate samples of viable cells were counted for each condition after trypsinization, with three repeats of counting for each Petri dish.

### Ferritin assay

MeT-5A cells were grown in 24-well plates and exposed for 24 and 48 h to 5 µg/mL of R-SWCNT, P-SWCNT, HP-SWCNT, or crocidolite fibres. The cells were rinsed twice with sterile phosphate-buffered saline (0.1 mM PBS, pH 7.4), then detached from the well by 0.25% trypsin (Sigma-Aldrich) containing 0.03% EDTA and pelleted. The pellet was washed with PBS (0.1 mM PBS, pH 7.4) and transferred to a microcentrifuge tube. 200 μL of M-PER reagent (Mammalian Protein Extration Reagent, Thermo Scientific) were added to each samples and shaken gently for 5 minute. After incubation, the lysates were collected and centrifuged at 14,000 × g for 10 minutes at 4 °C. The supernatants were transferred to a new tube for analysis. The ferritin concentrations in the lysates were measured using a Cobas® 6000 instrument (Roche Diagnostics USA, Indianapolis). Each data point represents the average of three measurements in at least three different experiments.

### X-Ray microscopy

To perform *in vitro* treatment experiments, MeT5-A cells were seeded at a concentration of 9 × 10^4^ cell/mL onto 100 nm thick silicon nitride (Si_3_N_4_) windows (Silson Ltd., Northampton, United Kingdom) contained in 24 multiwell plates. The day after seeding, the culture medium was replaced with fresh medium containing different nanomaterials at a concentration of 5 µg/mL, and MeT-5A cells were incubated at 37 °C for 24 h. MeT-5A cells similarly grown onto silicon nitride windows, but not exposed to nanomaterials (untreated cells), were used as control.

After incubation, samples were fixed at room temperature with 4% paraformaldehyde aqueous solution (Sigma Aldrich) for 20 minutes. Then, samples were washed with Milli-Q water before the analysis. For each experimental condition we selected 5 to 8 cells, grown on different silicon nitride windows, to be analysed by XRF microscopy.

In order to identify both the distribution of light and some heavy elements in the cells, we performed the experiments using soft X-rays (0.9 KeV) at the TwinMic beamline^44^ of Elettra synchrotron (Trieste, Italy) and harder X-rays (7.2 keV) at the ID21 beamline^45^ of ESRF synchrotron (Grenoble, France). In both cases the microscopes were operated in vacuum to allow the detection of light elements and reduce air absorption. In particular the harder X-rays are required for mapping Ca, S, P and Fe, whereas soft X-ray microscopy provides higher quality absorption and phase contrast images, together with elemental mapping of C, N and O. The X-ray absorption and phase contrast images outline the morphological features of the sample at sub-micrometer length scales, while the simultaneous acquisition of the XRF maps correlates the elemental distribution to the morphology.

Most of the experiments were performed at the TwinMic beamline^46^ of the ELETTRA synchrotron facility (ELETTRA, Trieste, Italy, www.elettra.trieste.it/twinmic) using the scanning X-ray microscopy (SXM) mode. In the SXM configuration the sample is raster-scanned with respect to a microprobe generated by Zone plate focusing optics. This TwinMic operation mode allows simultaneous monitoring of the absorption and phase contrast images in transmission by means of a configured CCD detector system^47,48^ and of the XRF emission by means of 8 Silicon drift detectors SDDs providing simultaneous information about the morphology and elemental distributions^49–51^. The experiments were carried out with a photon energy of 0.9 keV and a spot size of 450 nm, which was a good compromise for getting sufficient fluorescence signal for light elements (C, N, O) and for Fe. Prior XRF mapping, absorption and phase contrast images were acquired with a spot size of 250 nm to get higher resolution images of the analysed areas.

SR-XRF data were also acquired at the ID21 X-ray Microscopy beamline^45^ of the ESRF synchrotron facility (Grenoble, France). The samples were prepared with the same protocol used for the TwinMic experiments described above and were mapped for identifying elemental distributions^52^. A Ni coated double mirror deflecting in the horizontal plane ensured the harmonics rejection. A Si(111) double-crystal monochromator was used to select energy of 7.2 keV, above the Fe K absorption edge. The Kirkpatrick-Baez focusing mirrors focused the beam down to a micro-probe of 300 nm × 900 nm (V x H) with a photon flux of 5 × 10^10^ photons/s. The sample was raster scanned in the micro-beam to collect 2D fluorescence maps with a spot size of 500 nm. An SDD detector (Bruker, Germany) was used to detect the fluorescence photons emitted by the sample. Analysis of the SR-XRF images and spectra has been carried out by using the multiplatform program PyMCA^53^.

### Data availability

The datasets generated during and/or analysed during the current study are available from the corresponding author on reasonable request.

## Results and Discussion

### Microscopic characterization of SWCNTs

**Figure 1:**
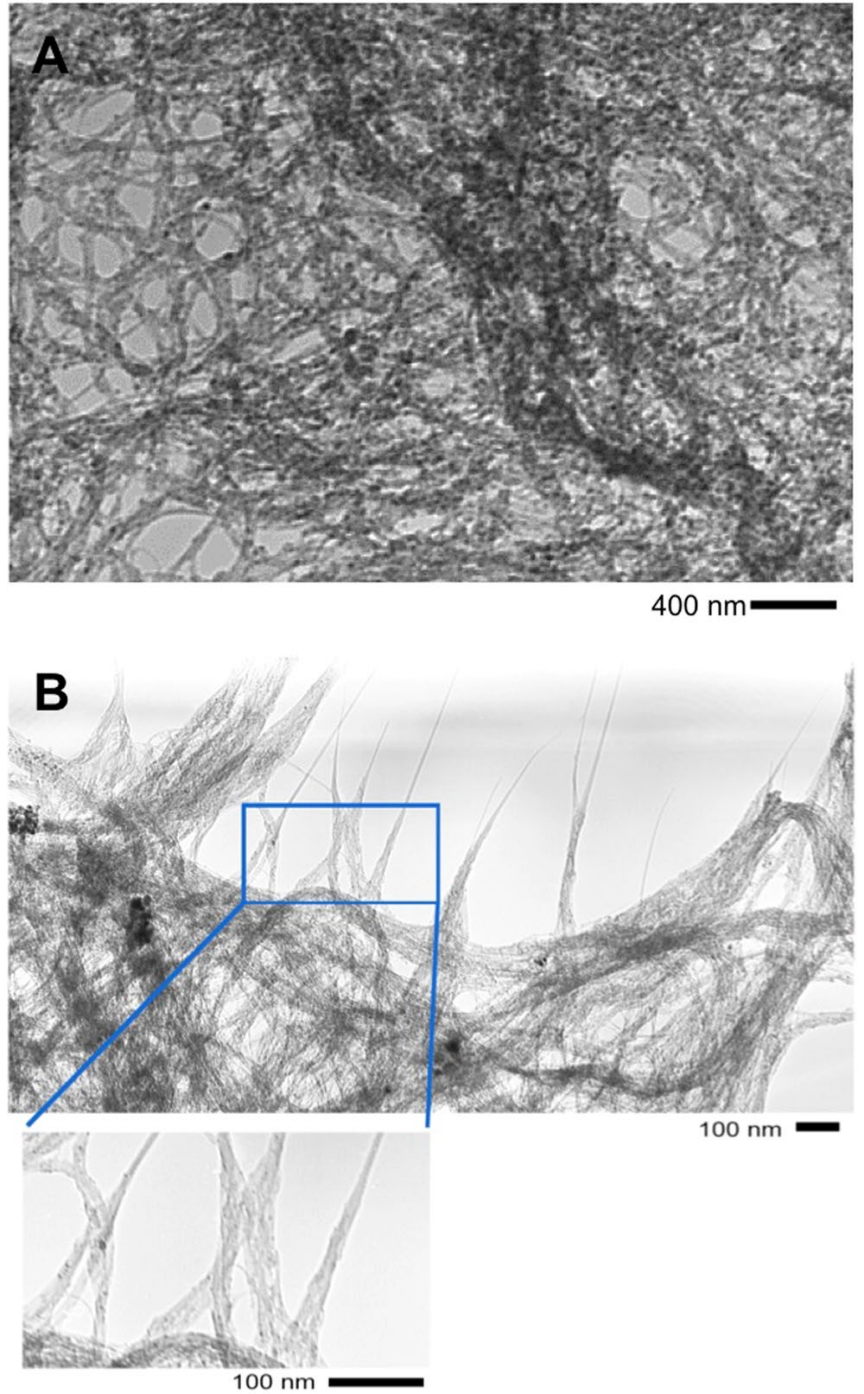
TEM images of raw (**A**) and purified (**B**) single-walled carbon nanotubes.

The results of TEM investigations of R-SWCNTs and P-SWCNTs are reported in Fig. 1. As expected, in line with the iron presence (Table 1), the images show several highly absorbing nanoparticles in the R-SWCNT preparation (panel a); particles are still present in the P-SWCNT preparation (panel b), even though at lower occurrence. As shown in Table 1 the iron content is drastically reduced in HP-SWNTs after the purification protocol, and not substantially visible as particles in TEM investigations (data not shown).

### Evaluation of cytotoxicity

MeT-5A cells viability tests were set at various concentrations of fibres (1 to 20 µg/mL) Figure S1 and the concentration of 5 µg/mL was selected in order to preserve a viability higher than 50% for the two most toxic materials (R-SWCNT and crocidolite) after 24 h 48 h of incubation, as shown in supporting information.

**Figure 2:**
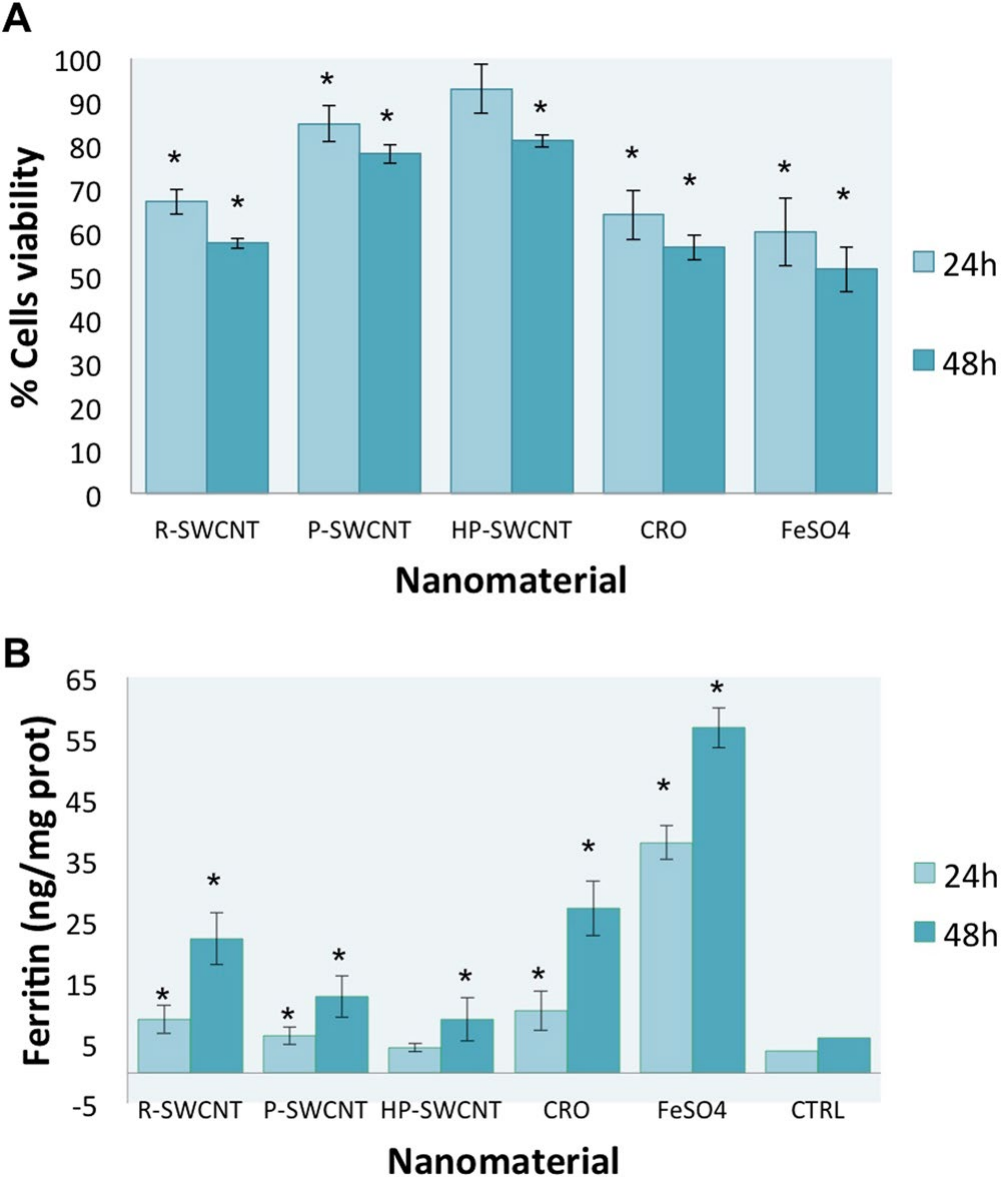
Viability test of cells after treatments. Panel (A) shows the toxic effects of nanomaterials on vitality of MeT-5A cells. The cells are grown for 24 h and then treated with nanomaterials at 5 *µ*g/ml for 24 h and 48 h. Relative cell viability was assessed by trypan blue dye exclusion method. Results are presented as percentage of living cells (*significantly reduced relative to control sample P < 0.05). Panel (B) shows the ferritin concentration of MeT-5A cells exposed to different nanomaterials (at the same conditions of viability test). Results are presented as nanogram of ferritin on milligram of total protein (*significantly increased relative to control sample P < 0.05).

The results, shown in Fig. 2A, reveal that after 24 h of incubation with R-SWCNT or crocidolite fibres the cell viability was around 64 and 65%, respectively, while decreasing to 58 and 56%, respectively, after additional 24 h of incubation. The exposure to a same concentration of P-SWCNT and HP-SWCNTs resulted in much better viability, clearly higher than 80%, both at 24 h and 48 h of treatment. It is interesting to note that the strong viability reduction obtained with raw carbon nanotubes and crocidolite fibres occurs similarly after incubating MeT-5A cells with iron sulphate (FeSO_4_) for 24 or 48 h.

Based on these results the concentration of 5 µg/mL and the incubation at 24 h for all nanomaterials was selected for X-ray fluorescence and microscopy analyses and ferritin assay.

### Ferritin assay

Ferritin assay was performed on cells exposed to the different nanomaterials in order to discriminate if the iron increase revealed by SR-XRF was due to its own release from nanomaterials or to a biochemical response of the cells. Ferritin is a protein that stores iron and releases it in a controlled amount in the body. The protein is maximally abundant in liver, spleen, bone marrow and macrophages, but it is also produced in pleural cells. The mesothelial cells are able to control the ferritin expression in an iron-dependent manner^34,54^. As shown in Fig. 2B, the exposure of MeT-5A cells to iron sulphate for 24 or 48 h produces a clear stimulation of ferritin production, with concentration values that are more than 10 times higher than control cells. The cells exposed to crocidolite fibres show ferritin levels that are from 3 to 10 times those of control cells, after 24 and 48 h of exposure, respectively. Notably, the cells treated with R-SWCNTs had a ferritin stimulation very similar to that of crocidolite (both at 24 and 48 h), suggesting a similar toxic response from the cell. Ferritin stimulation, although in much lower extent, is also registered in cells treated with P-SWCNTs, which contain less than 15% of iron impurities, while is negligible in cells treated with HP-SWCNTs (<5 wt% Fe).

### Synchrotron X-Ray Microscopy and Fluorescence: Iron increase in MeT-5A cells

**Figure 3:**
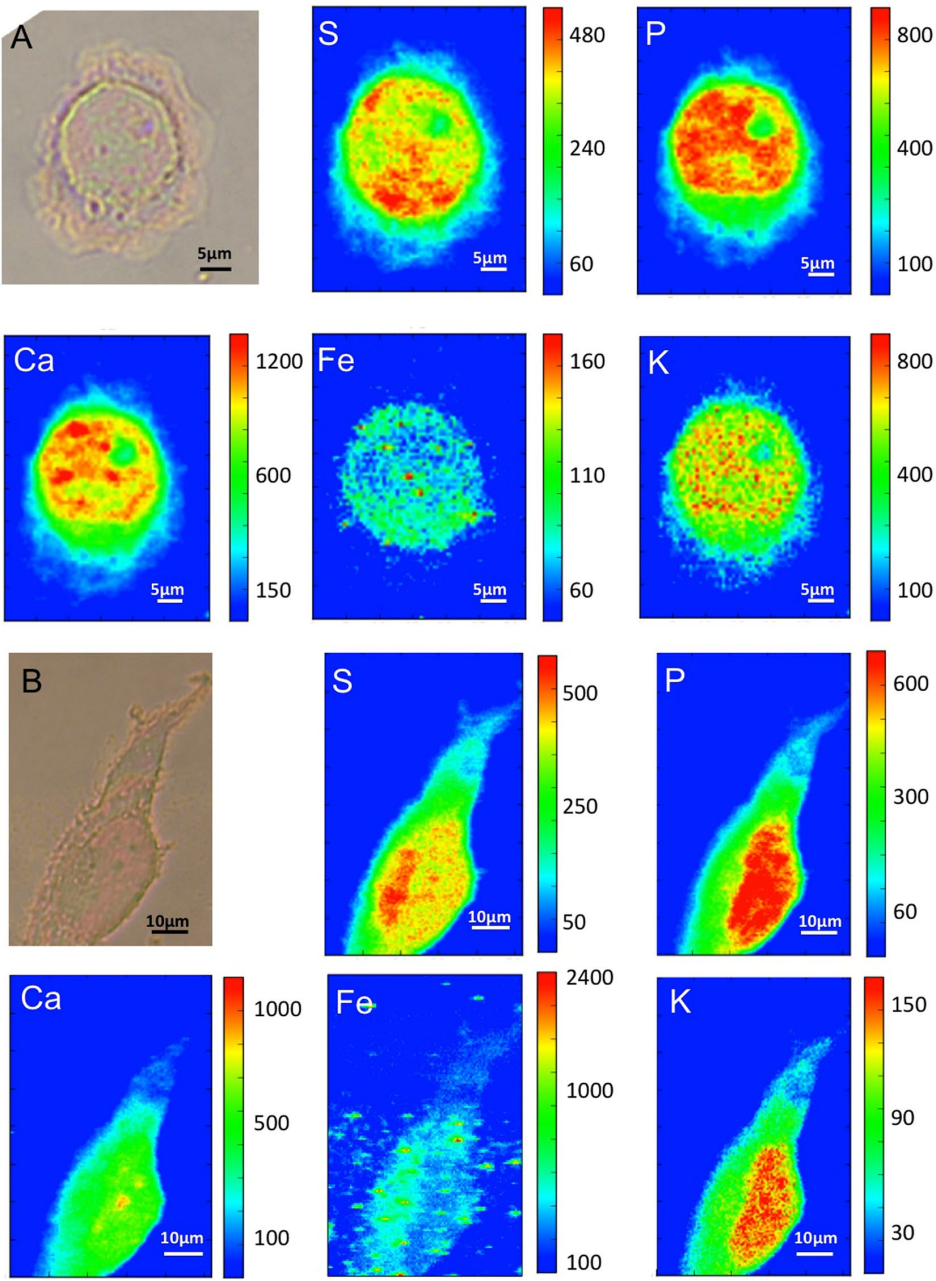
XRF and X-ray microscopy of untreated and treated cells. (**A**) Visible light image of the control cell (a) and the corresponding P, S, Ca, K, Fe XRF maps (32 µm × 42.5 µm) showing the distribution of different elements. (**B**) Visible light image of treated cell with FeSO_4_ (b) and the corresponding P, S, Ca, K, Fe XRF maps (52 µm × 60.5 µm). The XRF maps were acquired at ID21 beamline at 7.2 keV.

**Figure 4:**
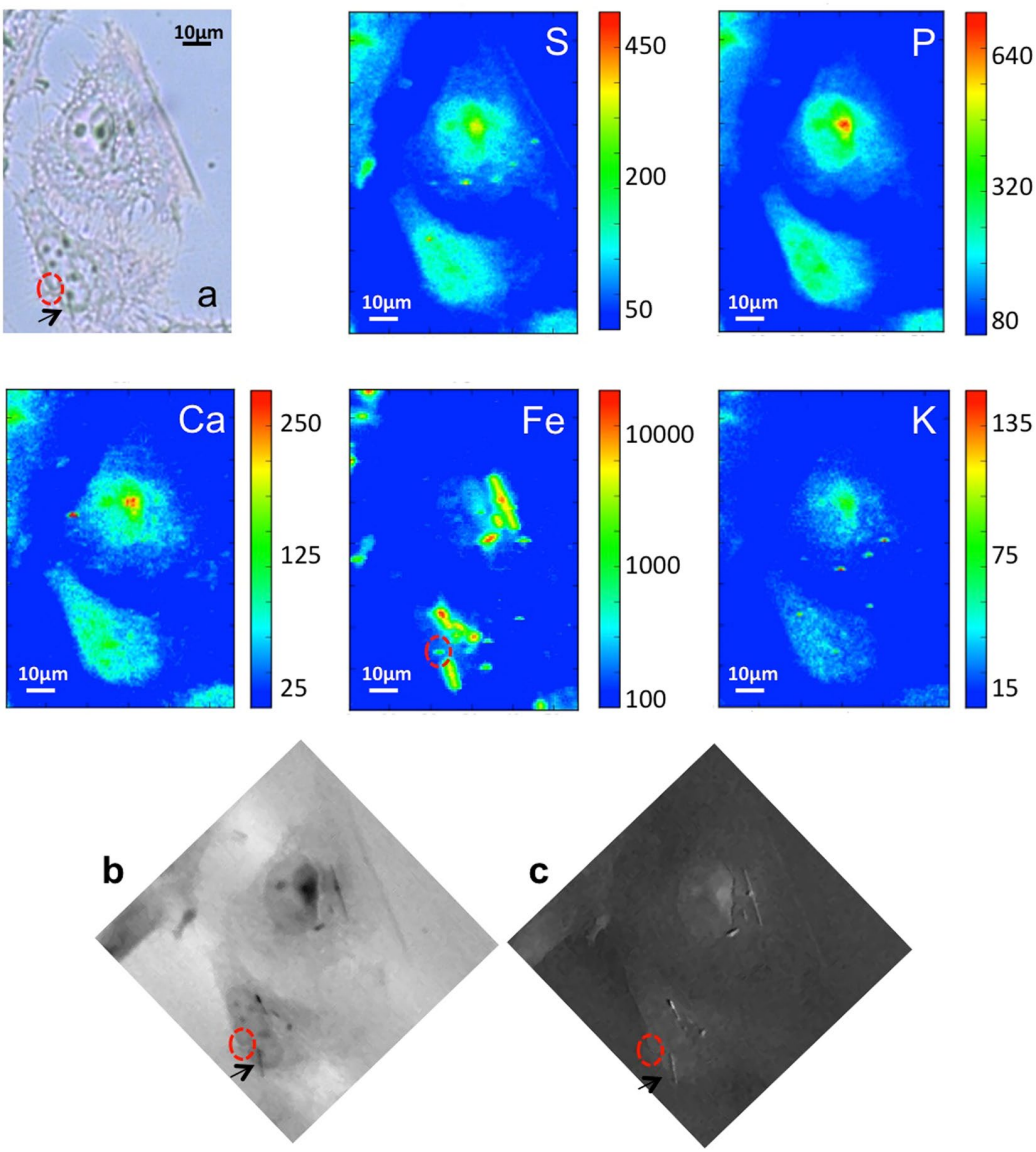
µXRF and X-ray microscopy of treated cells. Visible light image of two cells exposed to crocidolite fibres (**a**) and the corresponding P, S, Ca, K, Fe XRF maps (56 µm × 76.5 µm) showing the distribution of different elements. Fe map is displayed using a logarithmic scale. The bottom panels show the X-ray microscopy absorption (**b**) and phase contrast (**c**) images of the corresponding XRF maps. The absorption and phase contrast image were measured at the TwinMic beamline with photon energy 0.9 keV, whereas the XRF maps were acquired at ID21 beamline at 7.2 keV.

**Figure 5:**
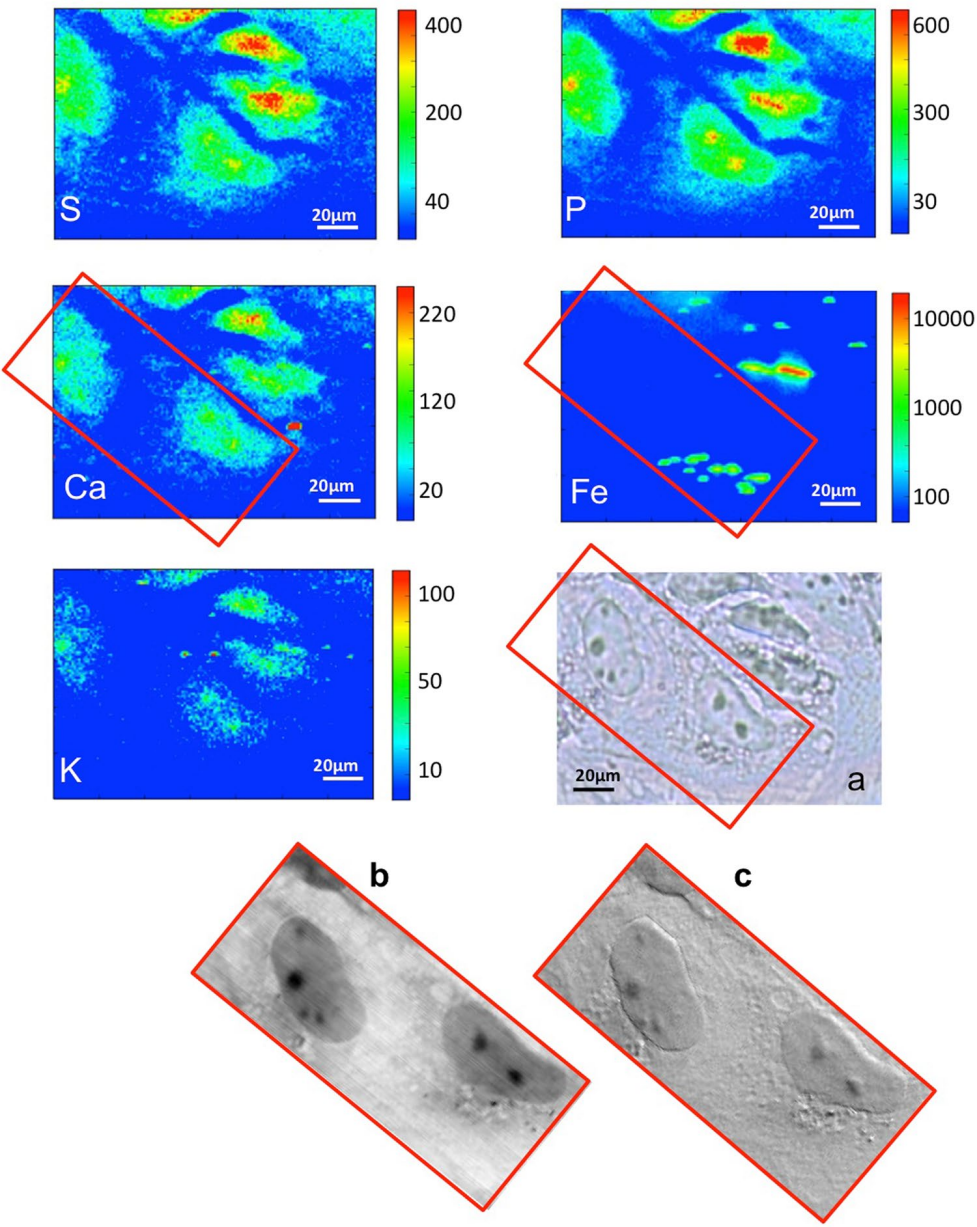
µXRF and X-ray microscopy of treated cells. Visible light image of two cells exposed to crocidolite fibres (**a**) and the corresponding P, S, Ca, K, Fe XRF maps (40 µm × 70 µm) showing the distribution of different elements. Fe map is displayed using a logarithmic scale. The bottom panels show the X-ray microscopy absorption (**b**) and phase contrast (**c**) images of two cells. The absorption and phase contrast images were measured at the TwinMic beamline with photon energy 0.9 keV, whereas the XRF maps were acquired at ID21 beamline at 7.2 keV.

After a selection under light microscopy, cells were raster scanned at the ID21 beamline of the European Synchrotron Radiation Facility (ESRF) using 7.2 keV photon energy. The previously described XRF set-up allows monitoring phosphorus, sulphur, calcium, potassium and iron distributions, as shown in Fig. 3. Panel A shows the elemental maps of a MeT-5A control cell: phosphorus (P) and sulphur (S) are constitutive elements of cells, and their distribution delineates the cell shape. In particular the P-rich zone in the central part of the cell can be attributed to the nucleus. Potassium (K) and calcium (Ca) show a rather homogeneous distribution. The counts for the iron fluorescence signal are low, indicating an overall low concentration of the element in healthy cells. Panel B shows a cell treated with 80 µM iron sulphate (FeSO_4_) for 24 h, used as control of intracellular increase of Fe concentration. The iron map reveals iron-rich regions, vesicles or aggregates, many of them extracellular. However, the logarithmic scale allows revealing a diffuse intracellular iron distribution that very likely derives from an iron uptake into the cells. Figures 4 and 5 show the analyses on cells exposed to crocidolite. The Fe maps reveal iron-rich segments that localize the crocidolite fibres, most of them seem intracellular as perceived by comparison with the optical image. In addition, the logarithmic scale allows revealing a diffuse intracellular iron increase that could derive partially from fibre dissolution (fibres contain about 30 wt% of iron^37^) and/or by an increased iron uptake into the cell. Compared to controls, cells exposed to asbestos show a reduced K presence, compatible with cell sufferance condition. Panels (b) and (c) of Figs 4 and 5 show the X-ray absorption and phase contrast images, respectively, of MeT-5A cells. The contrast of the X-ray images clearly indicates the different density between nucleus and other cell compartments (as nucleolus and cytoplasm). Moreover, soft X-ray microscopy allows to precisely reveal the presence of fibres not only in absorption mode, considering that the fibres are more absorbing than the surrounding cellular matter (b), but also in differential phase contrast (c). Indeed most of the fibres could not be seen in the optical image (a panels) since they are internalized. The combination of synchrotron X-ray fluorescence and soft X-ray microscopy allowed to study the fibres uptake in mesothelial cells and to distinguish the intracellular fibres from the extracellular ones. These techniques do not need staining or metal-coating procedures, in fact MeT-5A cells are only fixed, and they can be considered non-invasive analyses, which allow to investigate the morphology of cells and to reveal the cellular localization of the crocidolite fibres, by providing a better spatial resolution compared to the optical images and, therefore, much more detailed information.

For their needle-like shape the fibres possess an enhanced capacity to perforate the plasma membrane and only the smaller fibres are able to reach sub-cellular components of the cells up to nuclear regions. In Fig. 4b and c several small fibres are clearly visible, while not in (a), where they appear to be in contact with the nucleus or altering the perinuclear morphology (black arrows). In Fig. 5 the absorption and phase contrast images highlight a major morphological change of the cell caused by asbestos exposure. Soft X-ray microscopy allowed to reveal that numerous vesicles are present in the cytoplasm. After a careful analysis we have noticed that the iron-rich regions (shown in Fe map) are in proximity of these vesicles, which seem to be a cellular response to the nanomaterial toxicity. These morphological observations are in line with previous reports^55^.

**Figure 6:**
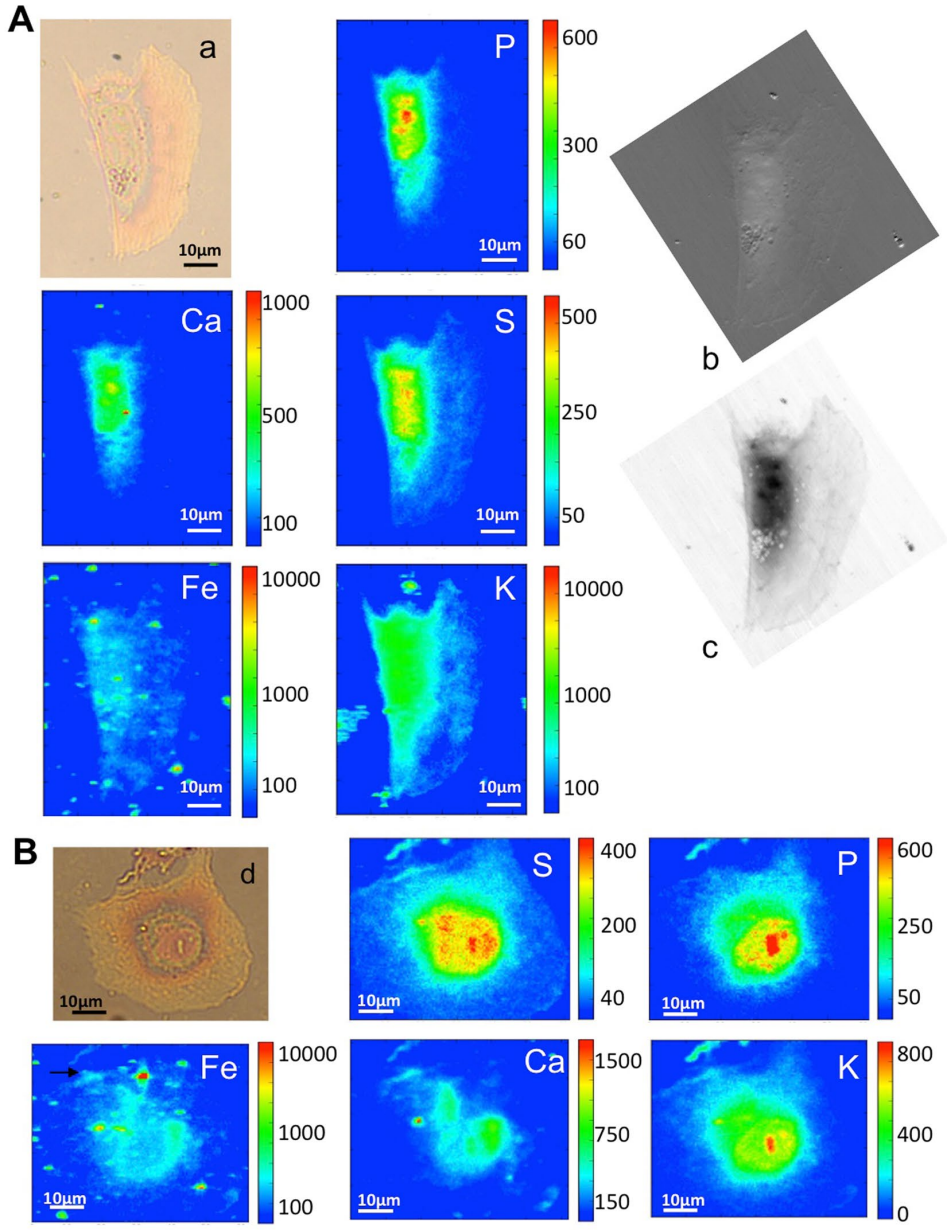
µXRF and X-ray microscopy of treated cells. (**A**) Visible light image of cell exposed to raw carbon nanotubes (R-SWCNT) (a) and corresponding P, S, Ca, K, Fe XRF maps (54 µm × 70.5 µm). The X-ray microscopy absorption (c) and phase contrast (b) images of the corresponding cell are reported to the side. Panel (B) Shows the visible image of another treated cells (d) and the corresponding P, S, Ca, K, Fe XRF maps (62 µm × 50.5 µm). Fe map is displayed using a logarithmic scale. The absorption and phase contrast images were measured at the TwinMic beamline with photon energy 0.9 keV, whereas the XRF maps were acquired at ID21 beamline at 7.2 keV.

Figure 6 shows the elemental maps of cells exposed to R-SWCNTs. Merging with the cellular shape (P and sulphur S maps), the Fe map reveals an overall iron increase and several iron-rich hot spots inside or surrounding the cells. Due to the pour solubility, it is highly possible that most of R-SWCNTs are aggregated and bundled, while only the smaller ones seem to interact with the plasma membrane, depositing along cellular edges. Bases on their dimensions and intensities, these iron-spots could be attributed to iron containing nanotube bundles or to residual iron particle aggregates detached from them. The iron content of R-SWCNTs is approximately 30 wt % (see Table 1). If we hypothesize that the iron impurities remain linked to the nanotubes, most of the spots localize with the bundles of R-SWCNTs. Moreover, in some cases R-SWCNT bundles seems to be internalized by mesothelial cells (as indicated by black arrow in Fe map of Fig. 6 panel B).

At some extent iron images are in line with the results obtained by other authors with a similar approach on macrophages exposed to CNTs^31^: in that study, XRF analyses revealed iron nanoparticles onto carbon nanotubes as well as detached iron nanoparticles inside cellular lysosomes. However, the authors affirm that CNTs exposure does not correlate to a ferritin increase, thus not causing an oxidative stress and toxicity^31^.

In our case, the iron impurities could be the cause of the reduction of cellular viability (as shown in Fig. 2), as well as of the clear alteration of iron metabolism inside cells. In line with the study performed on macrophages, our results are the first clear-cut evidence that this can happen in pleural cells. Although a lot of the iron nanoparticles seem to be extracellular as confirmed by rich iron-spots in Fe maps (Fig. 6 panels A and B), the homogenous iron increase inside the cell is clearly recognizable (the fluorescence signal levels are clearly higher than those of control cells) and is in agreement with the increase of ferritin production.

**Figure 7:**
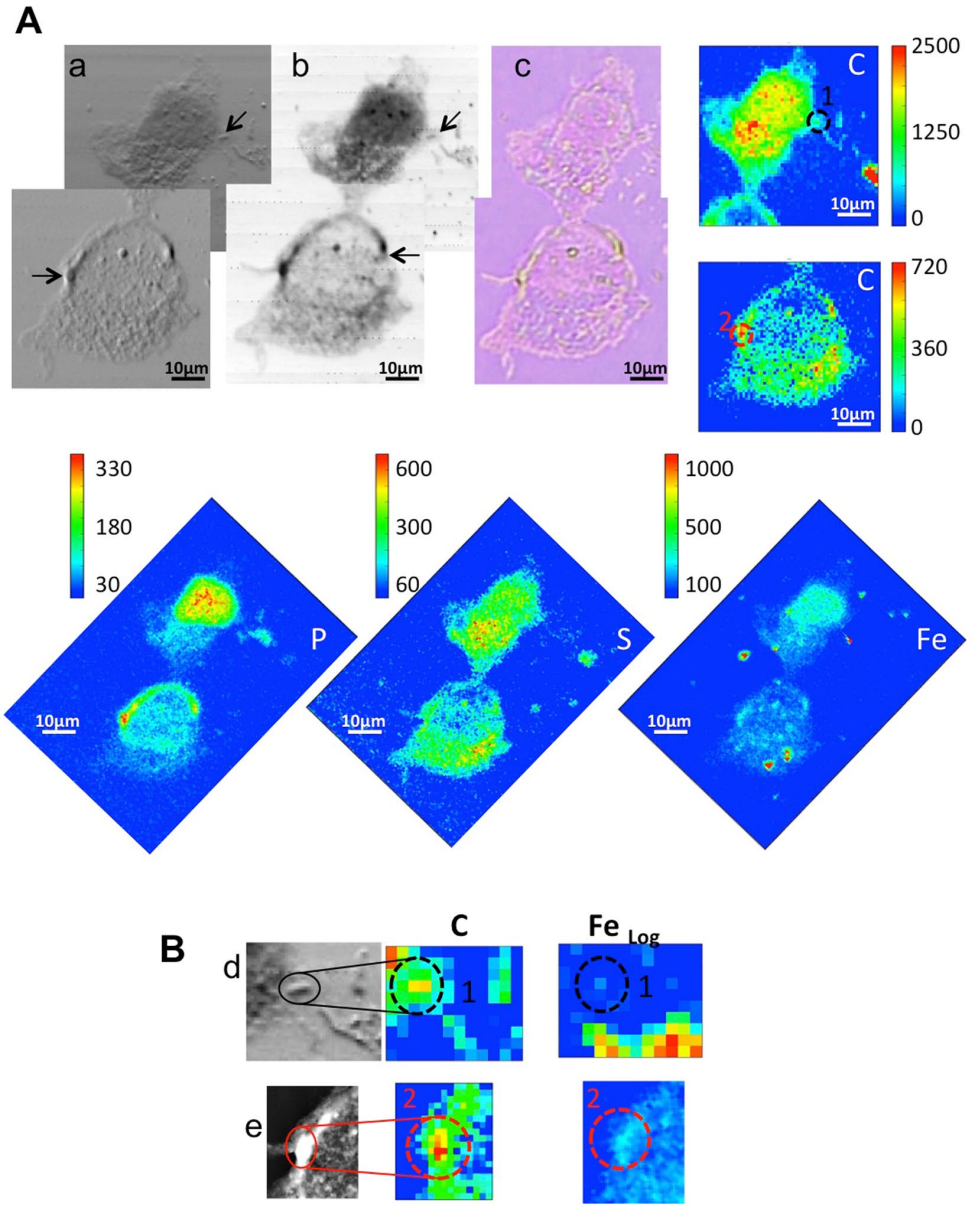
µXRF and X-ray microscopy of treated cells. (**A**) The X-ray microscopy absorption (a) and phase contrast (b) and visible light (c) images of exposed cells to purified carbon nanotubes (P-SWCNT). The C map, collected at the TwinMic beamline, is shown to the side. P, S and Fe maps, obtained at ID21 beamline, are reported below (52 µm × 73.5 µm). Panel (B) shows the specific localization of internalized P-SWCNT in a zoomed area of the absorption and phase contrast images of panel A (arrows in (b)). Phase contrast (d,e) images and corresponding C and Fe maps of zones indicated with the black and red circle. C and Fe maps confirm intracellular localization of carbon nanotubes in the region with the highest concentrations of both. Fe map is displayed using a logarithmic scale. The absorption and phase contrast images were measured at the TwinMic beamline with photon energy 0.9 keV, whereas the XRF maps were acquired at ID21 beamline at 7.2 keV.

It is interesting to note that in our case when the extracellular iron spots appear (outside of P and S distributions), they do not co-localize with any of the endogenous potassium and calcium. On the contrary, some co-localization is found intracellularly. The X-ray absorption (b) and contrast (c) images of Fig. 6 better resolve the morphology of the cell compartments, while the R-SWCNT are not recognizable, as expected from their small dimensions and low density. From the images a notable observation is the presence of small vesicles asymmetrically surrounding the nucleus, suggesting that the cell is in a severe status of sufferance. These morphological changes seem to differ from those caused by crocidolite exposure (Fig. 5), moreover iron appears uniformly increased in the cytoplasm, with some intense spots close but not clearly co-localizing with the small vesicles. Figure 7 (panel A) shows the X-ray absorption and contrast images and the corresponding XRF carbon maps (C) of cells exposed to P-SWCNTs. The X-ray absorption and contrast images [Panel A a) and b)] show an increased density in some areas, mainly ascribable to the higher thickness in the samples, in particular in the regions of P-SWCNT internalization (black arrow). This CNT internalization is also confirmed by carbon maps (panel B), where there is an increase of carbon level in correspondence of CNTs intracellular localization.

Figure 7 (panel A) shows also the phosphorus and sulphur maps that identify the cellular shape, while the iron map reveals several iron-rich spots inside or surrounding the cells. Similarly to Fig. 6, these iron-spots are compatible with iron containing nanotubes or to residual iron particles detached from them^31^. Also in the case of purified CNTs, containing less than 15 wt% of iron, the logarithmic scale allows revealing a diffuse intracellular iron increase inside the cells that could derive from increased Fe uptake and accumulation. As shown by Fig. 7, once inside the cells (panel B), the iron associated to CNTs bundles may be marginal, suggesting two hypothesis: i) that iron impurities can be released in the milieu (cellular and/or extracellular), and, ii) that the iron cellular content increases thanks to an increase uptake with concurrent increase of ferritin expression. While we do not have any evidence of CNTs compartmentalization in lysosomal or phagic vesicles, iron diffuse signal clearly suggest an overall increased uptake.

This suggests the occurrence of an alteration of the metal homeostasis in the exposed cells (evident both with R-SWCNTs and P-SWCNTs), similar to what seen in the case of asbestos^32^. This could be partially related to iron release from CNTs as well as from asbestos fibre dissolution^56^. In any case the high presence of iron in the cells is expected to trigger toxicity^57^. Iron is both an essential and a toxic element for biological systems. When not properly bound it can induce cell death by generating free radicals as it inter-exchanges between ferric and ferrous forms^58^. In addition pleural cells, as well as airway epithelial cells^59^, are not deputed to iron storage so accumulated iron would be eliminated with more difficulty, with cytotoxic effects as also demonstrated by viability tests (Fig. 2).

**Figure 8:**
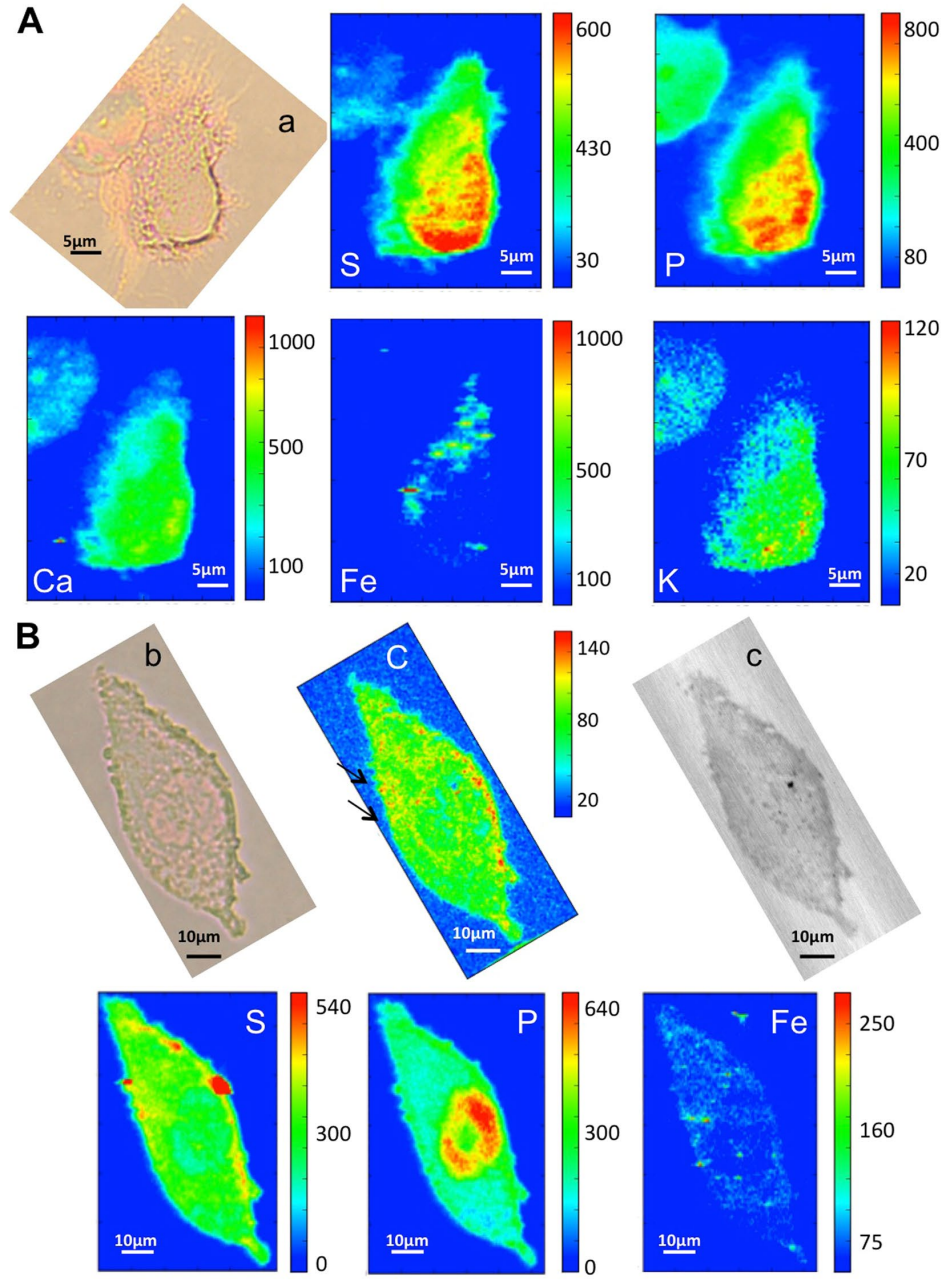
µXRF and X-ray microscopy of treated cells. (**A**) Visible light image of cell exposed to highly purified carbon nanotubes (HP-SWCNT) (a) and corresponding P, S, Ca, K, Fe XRF maps (36 µm × 46,5 µm) collected at ID21 beamline. Panel (**B**) shows visible light and absorption (b,c) images of another treated cell. The C map obtained at TwinMic beamline (0.9 keV) and S, P and Fe maps collected at ID21 beamline (7.2 keV) are also reported in the panel. Fe map is displayed using a linear scale.

The exposure of cells to HP-SWCNTs seems to not cause iron mediated toxicity, since iron content in cells is low. This is demonstrated both by viability (Fig. 2) and by results depicted in Fig. 8. Figure 8(A and B) shows the XRF maps of two cells exposed to highly purified HP-SWCNT. Looking at the iron map of panel A, one can note that there is a mild and inhomogeneous intracellular increase of the element; again the iron-spots can be linked to iron containing nanotubes or to residual iron particles detached from them, but they are clearly far fewer and less intense compared to the previous conditions (Figs 6 and 7).

Figure 8B shows the P and S maps that identify the cellular shape of another cell, while the iron map reveals several iron-rich spots inside the cell. The Carbon map seems to reveal a thickening of cellular borders suggesting the internalization of carbon nanotubes, in some cases further indicated by small iron spot (arrow in C map). The thickening is clearly visible in the absorption image as well. It is evident that in these cells exposed to highly purified carbon nanotubes (HP-SWCNT) there is a much lower effect on the iron metabolism, probably due to the higher purity grade of CNTs, which corresponds to a content of less than 5 wt% in iron. Actually it is necessary to mention that the different purification methodologies, performed using oxidative processes, introduce carboxylic groups mainly in correspondence of the tubes tips and of the defects. This oxidation processes allow not only to remove various percentage of metal nanoparticles, but also to obtain different degree of functionalization of the tubes themselves. In such way, also their solubility in aqueous media changes, being the lower for the R-SWCNT, increasing for P-SWCNT and being the better for HP-SWCNTs. Also this parameter could not be excluded in the different cytotoxicity behaviour of the analysed materials. In any case HP-SWCNTs are clearly less toxic (Fig. 2A) and seem not to alter significantly the ferritin expression after 24 h of incubation, with a marginal effect also after an incubation of additional 24 h (Fig. 2B).

## Conclusion

The present work demonstrates that the alteration of the iron metabolism, which is a key mechanism in asbestos toxicity, is also an important biological effect exerted by CNTs at cellular level. These findings represent a step forward in the understanding the potential toxicity of these carbon nanomaterials. In some previous CNT studies, bioavailable iron has been associated with increased oxidative stress^29^ and inflammatory responses^30^. Some types of nanoparticles have been already demonstrated to increase iron content in human airway epithelial cells^59^. A related alteration of ferritin content has been also provided in macrophages upon exposure to some pollutant^60^ as well as in airway epithelial cells exposed to nanoparticles or chrysotile^61^. Our study, for the first time, demonstrates that an alteration in the intracellular iron concentration takes place also in human pleural cells exposed to CNTs, together with an increase in ferritin production, although the steps that connect the iron increase to the cell toxicity need further clarifications. The effect could be attributed to the iron impurities present on CNTs and in a “dose-dependent” manner, implying that high purification procedures minimize this toxic effect. This evidence is extremely important to guide carbon nanomaterial production avoiding toxicity. On the other hand, evaluation of ferritin production as well as increased iron concentration measured by XRF in mesothelial cells model may represent a useful model and approach in nanotoxicology studies.

## Electronic supplementary material

Supplementary Information
