# Supplementary material for: Iron-related toxicity of single-walled carbon nanotubes and crocidolite fibres in human mesothelial cells investigated by Synchrotron XRF microscopy
Source: Sci Rep. 2018 Jan 15;8:706. doi: 10.1038/s41598-017-19076-1 (PMC5768674; doi:10.1038/s41598-017-19076-1)
Supplement: Supplementary file 1 — Supplementary Information [file 41598_2017_19076_MOESM1_ESM.pdf]

ORIGINAL

Short running header: Iron related toxicity of SWCNTs in MeT-5A cells by SR-XRF

Cammisuli F et al

Iron-related toxicity of single-walled carbon nanotubes and crocidolite fibres in human mesothelial cells investigated by Synchrotron XRF microscopy

Francesca Cammisuli[1], Silvia Giordani[2], Alessandra Gianoncelli[3], Clara Rizzardi[1], Lucia Radillo[1], Marina Zweyer[1], Tatiana Da Ros[4], Murielle Salomé[5], Mauro Melato[6], Lorella Pascolo[6]

[1]Department of Medical, Surgical, and Health Sciences, University of Trieste, 34149 Trieste, Italy.

[2]Department of Chemistry, University of Turin, Turin, Italy.

[3]Elettra - Sincrotrone Trieste, Basovizza, 34149 Trieste, Italy.

[4]Department of Chemical and Pharmaceutical Sciences, University of Trieste, 34149 Trieste, Italy.

[5]European Synchrotron Radiation Facility, 38000 Grenoble, Cedex 9, France

[6]Institute for Maternal and Child Health, IRCCS Burlo Garofolo, 34137 Trieste, Italy

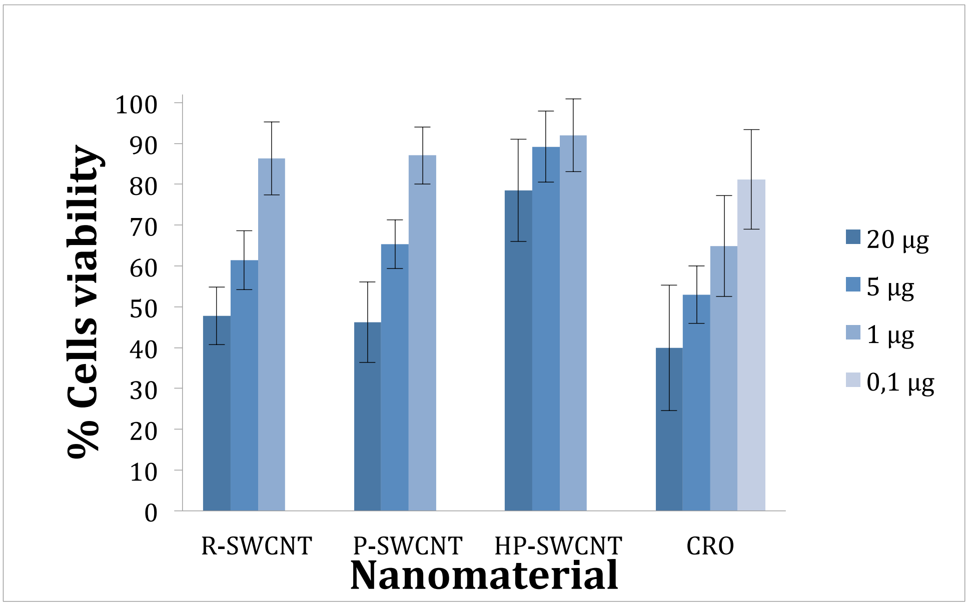

Figure S1. Viability test of cells after treatments. The panel shows the toxic effects of nanomaterials on vitality of MeT-5A cells. The cells are grown for 24h and then treated with nanomaterials at 20, 5 and 1 *μ*g/mL (and 0.1 for crocidolite) for 24h. Relative cell viability was assessed by trypan blue dye exclusion method. Results are presented as percentage of living cells.
